# Inhibition of Poxvirus Gene Expression and Genome Replication by Bisbenzimide Derivatives

**DOI:** 10.1128/JVI.00838-17

**Published:** 2017-08-24

**Authors:** Artur Yakimovich, Moona Huttunen, Benno Zehnder, Lesley J. Coulter, Victoria Gould, Christoph Schneider, Manfred Kopf, Colin J. McInnes, Urs F. Greber, Jason Mercer

**Affiliations:** a Institute of Molecular Life Sciences, University of Zurich, Zurich, Switzerland; b Institute of Biochemistry, ETH Zurich, Zurich, Switzerland; c MRC Laboratory for Molecular Cell Biology, University College London, London, United Kingdom; d Moredun Research Institute, Penicuik, United Kingdom; e Institute of Molecular Health Sciences, Department of Biology, ETH Zurich, Zurich, Switzerland; The Biodesign Institute, Arizona State University

**Keywords:** DNA replication, antiviral agents, poxvirus, vaccinia virus, viral transcription

## Abstract

Virus infection of humans and livestock can be devastating for individuals and populations, sometimes resulting in large economic and societal impact. Prevention of virus disease by vaccination or antiviral agents is difficult to achieve. A notable exception was the eradication of human smallpox by vaccination over 30 years ago. Today, humans and animals remain susceptible to poxvirus infections, including zoonotic poxvirus transmission. Here we identified a small molecule, bisbenzimide (bisbenzimidazole), and its derivatives as potent agents against prototypic poxvirus infection in cell culture. We show that bisbenzimide derivatives, which preferentially bind the minor groove of double-stranded DNA, inhibit vaccinia virus infection by blocking viral DNA replication and abrogating postreplicative intermediate and late gene transcription. The bisbenzimide derivatives are potent against vaccinia virus and other poxviruses but ineffective against a range of other DNA and RNA viruses. The bisbenzimide derivatives are the first inhibitors of their class, which appear to directly target the viral genome without affecting cell viability.

**IMPORTANCE** Smallpox was one of the most devastating diseases in human history until it was eradicated by a worldwide vaccination campaign. Due to discontinuation of routine vaccination more than 30 years ago, the majority of today's human population remains susceptible to infection with poxviruses. Here we present a family of bisbenzimide (bisbenzimidazole) derivatives, known as Hoechst nuclear stains, with high potency against poxvirus infection. Results from a variety of assays used to dissect the poxvirus life cycle demonstrate that bisbenzimides inhibit viral gene expression and genome replication. These findings can lead to the development of novel antiviral drugs that target viral genomes and block viral replication.

## INTRODUCTION

Viral infections are difficult to treat and prevent. Underlying technical reasons include diagnosis and viral persistence and the fact that viruses occur in large numbers, are genetically adaptable to environmental pressure, and are highly dependent on their hosts (1–3). This makes it challenging to treat virus infections with compounds that target viral factors such as enzymes or structural proteins. Compounds directed against host factors required for infection potentially endanger the host, although there is emerging evidence that clinically approved anticancer agents have significant efficacy against viruses in postexposure regimens (4).

Today's world population has become susceptible to poxvirus infection anew, after the discontinuation of smallpox vaccination over 30 years ago. Notable poxvirus cases include variola virus, the causative agent of smallpox, which despite eradication is ranked as a “category A pathogen” by the U.S. National Institute of Allergy and Infectious Diseases. Further agents include the vaccinia virus (VACV) Cantalago virus and cowpox viruses, which are contracted from infected animals and can cause fever and lesions (5–9), and monkeypox, which has a mortality rate estimated around 10% (10) and was responsible for the 2003 poxvirus outbreak in the United States (11, 12).

There are few current treatment options against orthopoxvirus infections. These include the live attenuated VACV-based vaccines Dryvax and ACAM2000, which can have some adverse effects, including fever, rash, encephalitis, and, in rare cases (1:1,000,000), death (13, 14). Small molecule compounds against orthopoxvirus infections include cidofovir, a nucleotide analog targeting the viral DNA polymerase (15), and ST-246 (tecovirimat [TPOXX]), the most promising antipoxvirus drug, which inhibits virus cell-to-cell spread (16–18). For both cidofovir and ST-246, poxvirus resistance has been reported (16, 19, 20). Remarkably, a single point mutation within the viral genome is sufficient to give rise to ST-246 resistance (16). In the face of the limited number of antipoxvirus drugs, there is an obvious need for novel antivirals directed against poxviruses.

Targeting of the viral replication machinery by antivirals has been successfully employed against RNA and DNA viruses (15, 21–24). Inhibition of viral polymerases and helicases is effective as this strategy leads to sustained inhibition of genome replication, thereby slowing the emergence of drug resistance mutations. To date, direct targeting of viral genomes by antiviral agents has not been reported.

Bisbenzimides are a class of fluorescent dyes that bind within the minor groove of double-stranded DNA (dsDNA), preferentially to AT-rich regions (25–29). These compounds have been used to drive proapoptotic and cytostatic activity in cancer cells (30, 31). In addition, bisbenzimide derivatives have been reported to indirectly modulate mammalian and bacterial topoisomerase I and II activity (32–35). Yet their application as antiviral agents has not been explored.

Here, we present evidence that a set of bisbenzimide derivatives that are commonly known as Hoechst compounds (29, 36–38), display potent antipoxvirus activity far separated from cell toxicity. Dissection of poxvirus temporal gene expression, uncoating, genome replication, and virus yield indicates that bisbenzimide-mediated antipoxvirus activity occurs through inhibition of viral intermediate (IG) and late (LG) gene transcription as well as genome replication.

## RESULTS

### Bisbenzimides inhibit VACV infection.

**FIG 1:**
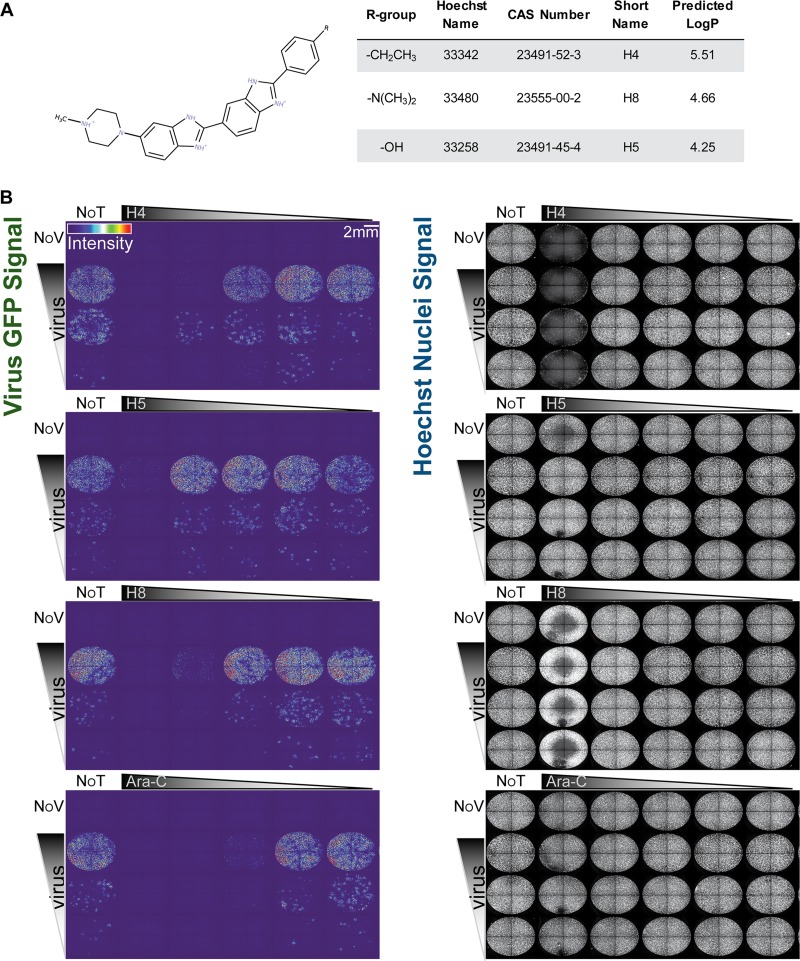
(A) Chemical structure, properties including partitioning coefficient (LogP), and compound information of the bisbenzimides used in this study. (B) Bisbenzimides (H4, H5, and H8) block VACV replication in tissue culture. BSC40 cells were infected with a serial dilution of E/L EGFP VACV and treated with serial dilutions of H4, H5, H8, or AraC. Full-well images show EGFP-expressing infected cells color coded by intensity (left panels). Nuclei were detected by staining with Hoechst (right panels). Experiments were performed in triplicate; representative images are displayed. NoT, not drug treated; NoV, not virus infected.

**FIG 2:**
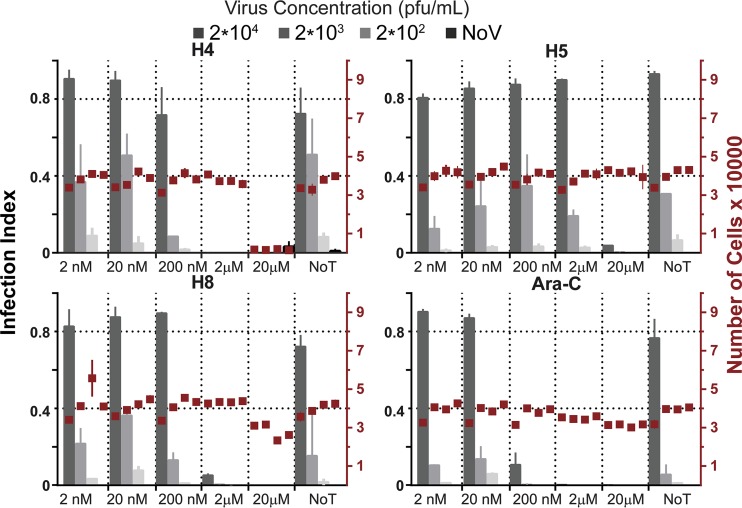
Quantification of infected EGFP-expressing cells and total cell number (nuclei) from Fig. 1B. Gray bars indicate the infection index, and red boxes indicate the cell number for each condition tested. Experiments were performed in triplicate. Results are displayed as means ± standard deviations (SD).

To determine if bisbenzimides have antiviral activity against VACV, we tested the abilities of three different bisbenzimides to inhibit viral plaque formation assays: Hoechst 33342 (H4), Hoechst 33258 (H5), and Hoechst 34580 (H8) (Fig. 1A). Confluent monolayers of African green monkey kidney (BSC40) cells were infected with serial dilutions of the VACV strain Western Reserve (WR), which expresses enhanced green fluorescent protein (EGFP) from an early/late promoter (WR E/L EGFP) in the presence or absence of H4, H5, or H8. A known inhibitor of VACV DNA replication, cytosine arabinoside (AraC) (39, 40), served as a positive control in these experiments. At 24 h postinfection (hpi), plates were imaged for nuclei, indicating cell numbers, and GFP expression, a surrogate for infection (Fig. 1B). The total cell number and the number of infected cells were quantified using Plaque 2.0 (41) (Fig. 2). The Hoechst compounds displayed no apparent cell toxicity, with the exception of H4 and, to a slight extent, H8, at the highest concentration tested, 20 μM. With each Hoechst compound, dose-dependent inhibition of VACV infection was observed. H4 was the most potent compound, causing a complete block of VACV infection at 2 μM regardless of virus concentration. H5 and H8 were less effective, both reaching complete inhibition at 20 μM (Fig. 2). These effects were not specific to BSC40 cells, since similar results were obtained in L929 mouse fibroblasts (data not shown). The data show that the bisbenzimides H4, H5, and H8 exert potent inhibitory activity against VACV infection with little cell toxicity when used at low micromolar concentrations.

### Bisbenzimides block intermediate and late but not early viral gene expression.

We next tested the impact of the most potent early/late viral gene expression inhibitor (H4) and the least toxic compound (H5) on early and late viral gene expression. VACV gene expression occurs in three temporal stages: early, intermediate, and late. Early gene expression (EGE) occurs prior to DNA replication, while intermediate gene expression (IGE) and late gene expression (LGE) require DNA replication.

**FIG 3:**
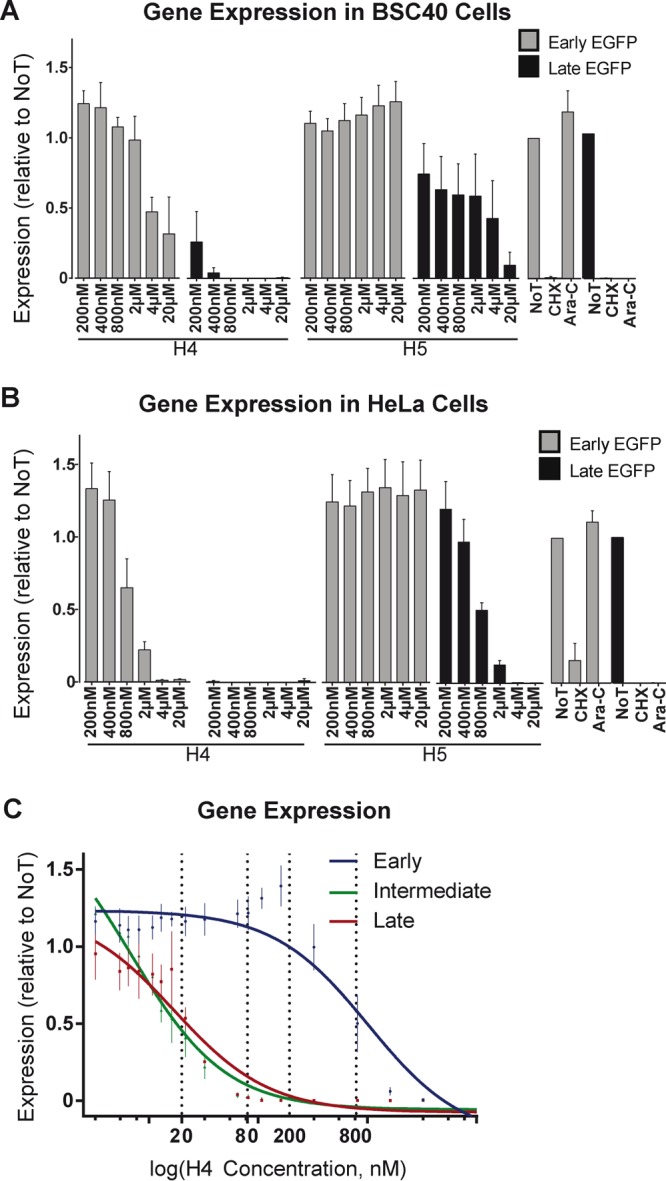
Bisbenzimides inhibit VACV intermediate and late gene expression. (A and B) BSC40 (A) or HeLa (B) cells were infected with WR E EGFP (gray bars) or WR L EGFP (black bars) VACV. Cells were scored for EGFP expression by flow cytometry, and infected cells were quantified relative to untreated cells. CHX and AraC served as controls for these experiments. (C) HeLa cells treated with various concentrations of H4 were infected with WR E EGFP (blue line), WR I EGFP (green line), or WR L EGFP (red line), and the percentage of EGFP-positive infected cells was quantified by flow cytometry. These values were fitted to dose-response curves to estimate EC_50_ and EC_90_ values (dashed lines).

BSC40 or HeLa cells were pretreated with H4 or H5 and infected with recombinant VACVs encoding EGFP under the control of an early (WR E EGFP) or late (WR L EGFP) viral promoter. Cells treated with cycloheximide (CHX) and AraC served as positive controls for inhibition of EGE and LGE, respectively (Fig. 3). Infection was quantified by flow cytometry 8 hpi. In BSC40 cells, H4 treatment inhibited EGE up to 42% at 20 μM and completely blocked LGE at 800 nM and above. H5 on the other hand did not inhibit EGE at any of the tested concentrations but showed a dose-dependent inhibition of LGE of up to 90% from 2 μM to 20 μM (Fig. 3A). In HeLa cells, the trends of inhibition by H4 and H5 were similar, but the compounds were more potent than in BSC40 cells, with H4 resulting in complete inhibition of EGE at 4 μM and LGE at 200 nM and with H5 showing no impact on EGE but inhibiting LGE by 100% at 4 μM (Fig. 3B).

The half-maximum and maximum effective concentration (EC_50_ and EC_90_, respectively) for inhibition of EGE, IGE, and LGE were determined for H4 (Fig. 3C). HeLa cells were pretreated with various concentrations of H4 and infected with WR E EGFP, WR I EGFP, or WR L EGFP viruses. Consistent with the data in Fig. 2, the EC_50_ of H4 for EGE was 800 nM and the EC_90_ was 1.6 μM (Fig. 3C, blue line). H4 was even more effective against IGE and LGE, with EC_50_ inhibition of IGE or LGE at 20 nM and EC_90_ at 80 nM (Fig. 3C, green and red lines). These results show that the bisbenzimide H4 is an effective inhibitor of VACV IGE and LGE at nanomolar concentrations.

### H4 blocks VACV plaque formation and reduces viral yield.

**FIG 4:**
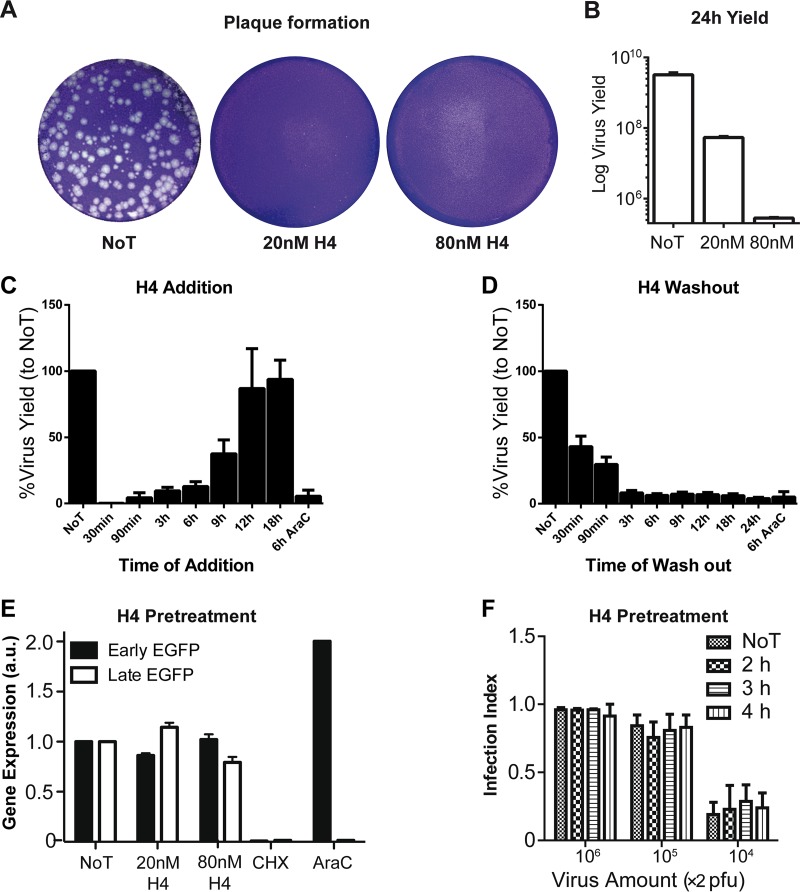
H4 inhibits plaque formation, reduces virus yield, and blocks early VACV infection without impacting particle infectivity. (A) HeLa cells were infected with 150 PFU of WT VACV, and infection was allowed to proceed for 72 h. The plates were stained with crystal violet to visualize plaques. (B) HeLa cells were infected with WT VACV (MOI of 1). Twenty-four hours postinfection, cells were harvested and lysed and virus yield was determined by titration and plaque formation. (C) HeLa cells were infected with WT virus (MOI of 1), and 80 nM H4 was added at the indicated time points. A sample subjected to AraC addition at 6 hpi was included as a positive control for inhibition. Cells were harvested 24 hpi, and virus yield was determined for each sample by serial dilution plaque assay. (D) HeLa cells were infected with WT virus (MOI of 1) in the presence of 80 nM H4. At the indicated time points, cells were washed, and infection was allowed to proceed. A sample subjected to AraC washout at 6 hpi was included as a positive control for inhibition. At 24 hpi, virus yield was determined for each sample by serial dilution plaque assay. (E) WR E EGFP (black bars) or WR L EGFP (white bars) virions were preincubated with 20 or 80 nM H4 for 30 min at room temperature. Virus particles were washed three times and used to infect HeLa cells. Samples were analyzed by flow cytometry for infected EGFP-positive cells at 6 hpi (early) and 8 hpi (late). a.u., absorbance units. (F) WR E/L EGFP virions were preincubated with 2 μM H4 for 2, 3, or 4 h. Virions were washed and used to infect HeLa cells prior to fixation and analysis by Plaque 2.0 for total nuclei and EGFP-positive infected cells. (A to F) All experiments were performed in triplicate; representative images are shown (A), or results are displayed as means ± SD (B to F).

Given the potent effect of H4 on IGE and LGE, we next assessed the ability of H4 to inhibit plaque formation and produce progeny. For plaque formation, monolayers of HeLa cells were infected with 150 PFU of VACV in the absence (not drug treated [NoT]) or presence of 20 or 80 nM H4. At 72 hpi, monolayers were assessed for plaque formation by staining the residual cells with crystal violet (Fig. 4A). In the presence of 20 nM H4, both the size and number of plaques were strongly reduced, with a few small plaques detected. At 80 nM H4, no visible plaques were detected.

Next we assessed the impact of H4 on virus production in HeLa cells. In the presence of 20 nM H4, the number of infectious particles was reduced by 1.5 logs at 24 hpi. In the presence of 80 nM H4, virus production was reduced by 4 logs relative to the 24-h yield in untreated cells (Fig. 4B). The results show that H4 effectively blocked VACV plaque formation and the production of infectious particles, even over extended periods (72 h) of incubation.

### H4 targets an early stage of VACV infection.

To address the stage of the virus life cycle blocked by H4, we conducted add-in or washout experiments with H4 at different times of infection. When 80 nM H4 was added as early as 6 hpi, virus yield was reduced by ≥90% compared to that in the cells not drug treated (NoT) (Fig. 4C). Addition of H4 at 9 hpi gave a 50% reduction, and addition at 12 hpi had no impact on virus yield (Fig. 4C). In washout experiments, only early washout of H4—for example, at 30 or 90 min postinfection—partially rescued virus yield, whereas washout at later times essentially had no rescue effects (Fig. 4D). These results indicated that H4 is most effective during early stages of infection.

### Pretreatment of purified VACV virions with H4 does not impact infectivity.

Remarkably, H4 had little impact on EGE but diminished virus yield at early infection times, when events such as EGE occur. To resolve this puzzling notion, we tested if H4 directly affected the infectivity of virions. WR E EGFP and WR L EGFP viruses were preincubated with H4 at 20 nM (EC_50_) or 80 nM (EC_90_), extensively washed to remove residual bisbenzimide, and added to HeLa cells. Cells were harvested, and infection was analyzed by flow cytometry 8 hpi (Fig. 4E). The results showed that preincubation of virions with H4 had no significant impact on either EGE or LGE. To confirm these results, a range of WR E/L EGFP virus concentrations were preincubated for various times with 2 μM H4, a concentration that completely blocked EGE and LGE in HeLa cells (Fig. 3B and C). Untreated (NoT) and pretreated virions were washed extensively before addition to cells. After 24 h, infection was quantified using the microscopy-based Plaque 2.0 assay (Fig. 4F). As expected, infection was dose dependent, yet even at 2 μM H4, no inhibition of VACV infection was observed. These results show that H4 does not directly impact the infectivity of extracellular virions but rather acts on a critical intracellular stage of the VACV life cycle.

### H4 treatment does not impact VACV genome uncoating.

Given that H4 had no impact on EGE but effectively blocked IGE and LGE, we assessed genome uncoating (reviewed in references 42 and 43), as it is a prerequisite for VACV genome replication and subsequent IGE and LGE. Incoming VACV genomes released into the cytoplasm, termed prereplication sites, can be visualized by immunofluorescence directed against the viral single-stranded DNA binding protein I3, or with click chemistry-based detection of single virus genomes (44–48).

**FIG 5:**
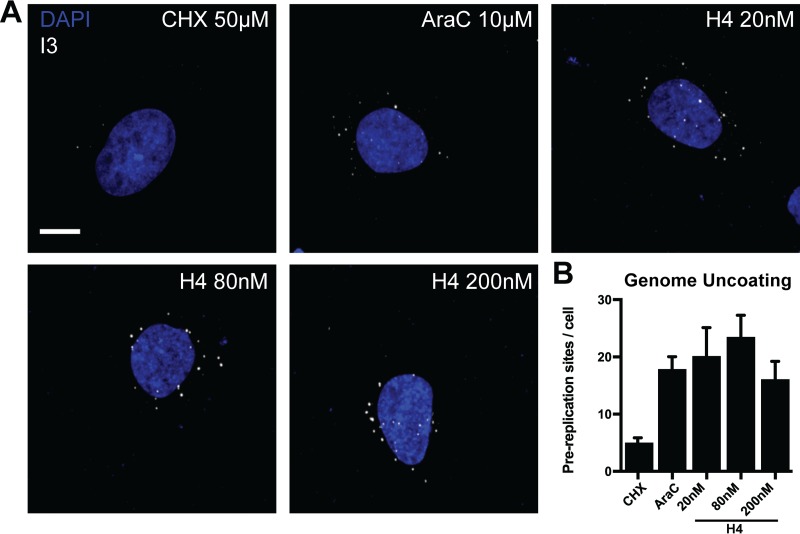
H4 does not impact viral genome uncoating. (A) HeLa cells were infected with WT VACV (MOI of 10) in the presence of 20, 80, or 200 nM H4 and AraC. Prereplication sites were visualized by immunofluorescence staining against I3 followed by confocal microscopy. CHX and AraC served as controls for uncoating and replication, respectively. (B) Quantification of prereplication sites per cell from panel A. (A and B) Experiments were performed in triplicate; representative images are displayed, and results are shown as means ± standard errors of the means (SEM). Scale bar, 10 μm.

Here we used I3 staining to test the influence of H4 on VACV prereplication site formation. HeLa cells were infected in the presence of 20, 80, or 200 nM H4 and AraC, fixed at 5 hpi, and stained for I3 (Fig. 5A). AraC, which blocks viral replication post-uncoating, and CHX, which blocks uncoating by inhibiting the synthesis of the VACV uncoating factor (47), were included as controls. As expected, CHX prevented the formation of prereplication sites, while AraC did not affect the number of I3 puncta. H4 did not affect the number of I3-positive prereplication sites (Fig. 5B). These results show that H4 does not affect VACV genome uncoating even at concentrations above those that inhibit IGE and LGE.

### High concentrations of H4 inhibit VACV DNA replication.

**FIG 6:**
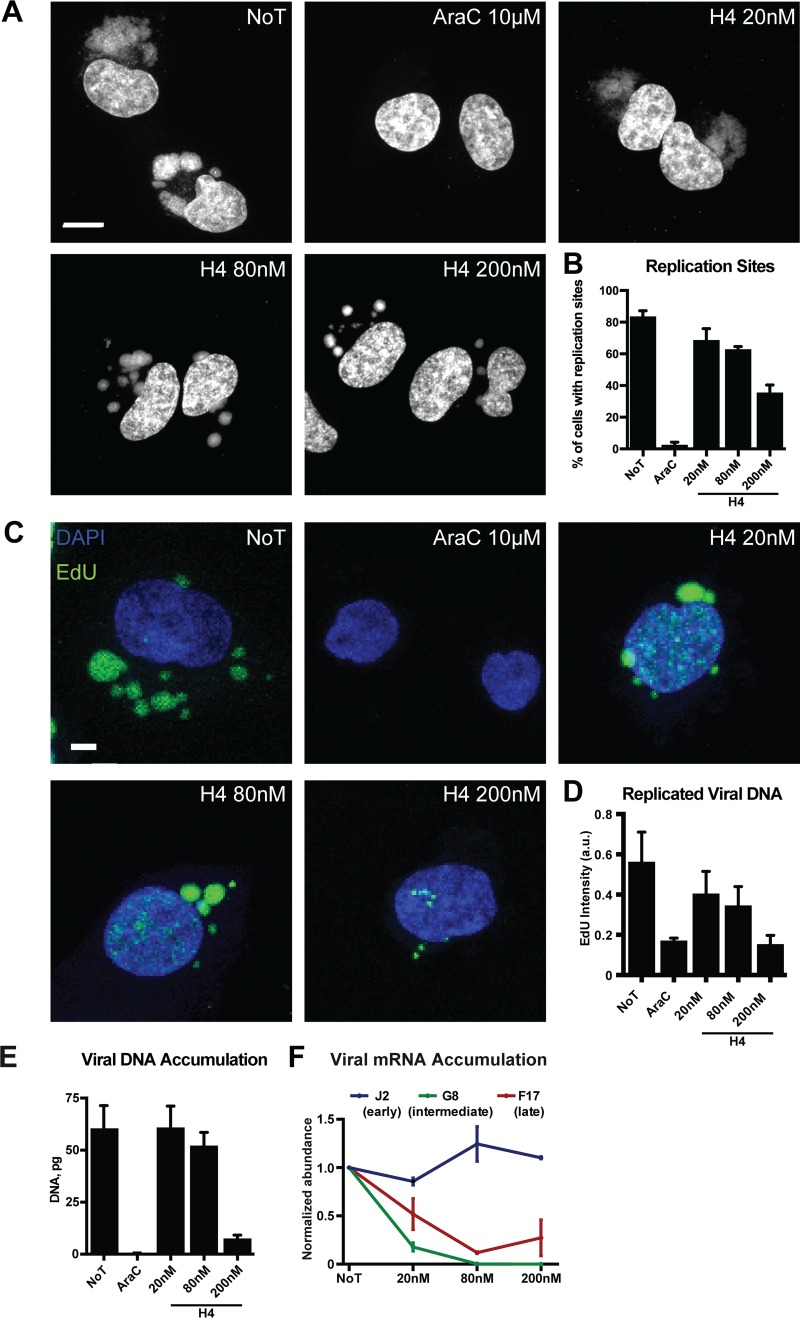
H4 attenuates VACV IG/LG transcription and DNA replication in a dose-dependent fashion. (A) HeLa cells were infected (MOI of 10) in the presence of 20, 80, or 200 nM H4. At 8 hpi, cells were fixed and stained with DAPI and imaged by confocal microscopy. Scale bar, 10 μm. (B) The number of cells with cytoplasmic replication sites was quantified per condition. AraC served as a control for inhibition of DNA replication site formation. (C) HeLa cells were infected (MOI of 10) in the presence of 20, 80, or 200 nM H4 and EdU. At 8 hpi, EdU incorporation was detected with a Click-iT EdU imaging kit followed by confocal microscopy. Scale bar, 10 μm. (D) The total intensity of EdU incorporation into replication sites was quantified. (E) The amount of viral DNA from cells infected in the absence or presence of H4 at different concentrations was quantified by qPCR at 8 hpi. AraC served as a control for inhibition of DNA replication. (F) The levels of early (J2), intermediate (G8), and late (F17) viral mRNA from infected HeLa cells were quantified by RT-qPCR. Cells were infected in the absence or presence of various concentrations of H4, and RT was performed at 2 hpi for J2, 4 hpi for G8, and 8 hpi for F17. Results are displayed as the average abundance normalized to untreated samples. All experiments were performed in triplicate; representative images (A and C) or means ± SD (B and D to F) are displayed.

We next tested the impact of H4 on VACV DNA replication site formation. Cells were infected in the presence of 20, 80, or 200 nM H4 and assessed for replication site formation by staining with 4′,6-diamidino-2-phenylindole (DAPI) at 8 hpi. As expected, untreated cells contained large cytoplasmic VACV replication sites, while AraC-treated cells had none (Fig. 6A). The replication sites in H4-treated cells showed phenotypic differences from control infections. At 20 nM, the replication sites appeared slightly smaller and more diffuse, and at 80 nM and 200 nM, the size of the replication sites was reduced and many small DAPI-positive puncta were seen (Fig. 6A). When the number of cells containing replication sites was quantified, without accounting for their size or number, treatment with 20 or 80 nM H4 showed 14.9% and 20.7% decreases, respectively, while treatment with 200 nM H4 decreased the number of cells containing replication sites by 48.2% (Fig. 6B). We noticed that the replication sites seen in the presence of 200 nM H4 were larger than the prereplication sites seen in the presence of AraC (Fig. 5A). This suggested that H4 did not block DNA replication initiation, but rather acted after the onset of replication.

To assess the impact of H4 on ongoing replication we used 5-ethynyl-20-deoxyuridine (EdU) and click chemistry labeling (48). Cells were infected in the absence or presence of 20, 80, or 200 nM H4, and the incorporation of EdU into viral replication sites was assessed at 8 hpi (Fig. 6C). Untreated cells displayed numerous bright EdU-positive replication sites. At 20 and 80 nM, the replication sites appeared less numerous but brighter, while at 200 nM, small replication sites with little EdU incorporation were seen (Fig. 6C). Quantification of the intensity of EdU per cell confirmed that nucleoside incorporation into viral replication sites was slightly reduced at 20 and 80 nM H4 and strongly reduced in the presence of 200 nM H4 to the levels seen in the presence of AraC (Fig. 6D).

### H4 blocks VACV DNA replication and IGE and LGE in a dose-dependent fashion.

Given the considerable size of the viral replication sites seen in the presence of 20 and 80 nM H4, we performed quantitative PCR (qPCR) to quantify VACV DNA synthesis in the presence of H4 (49). Total DNA was extracted from cells infected with VACV at 8 hpi, and the amount of viral DNA was quantified. While AraC blocked DNA accumulation as expected, no defect in viral DNA content was seen in the presence of 20 nM H4, and a modest decrease of 11% was observed at 80 nM H4 (Fig. 6E). VACV DNA accumulation was reduced by 88% in the presence of 200 nM H4, consistent with the small replication site phenotype observed in Fig. 6.

As VACV IGE and LGE occur after DNA replication, it was surprising to find that DNA accumulation was largely unimpeded in the presence of 20 and 80 nM H4, the respective EC_50_ and EC_90_ against VACV infection. We reasoned that H4 may be inhibiting transcription of VACV IGE and LGE at these low concentrations. Using quantitative reverse transcription-PCR (RT-qPCR), we assessed the impact of H4 on the accumulation of viral early (J2), intermediate (G8), and late (F17) mRNAs (Fig. 6F). While H4 had little impact on early viral mRNA amounts, accumulation of intermediate mRNA was diminished by 72% and that of late mRNA by 48% at 20 nM H4 (Fig. 6F). At 80 and 200 nM H4, intermediate mRNA accumulation was completely abrogated, and late mRNA was reduced by 88 and 76%, respectively. These results indicate that the bisbenzimide H4 impedes VACV infection by inhibiting two stages of the virus life cycle: at low levels (20 nM and 80 nM), H4 inhibits IG and LG transcription, and at elevated levels (200 nM), it inhibits VACV DNA replication as well as IG and LG transcription.

### Poxvirus infection is acutely sensitive to the antiviral activity of H4.

**FIG 7:**
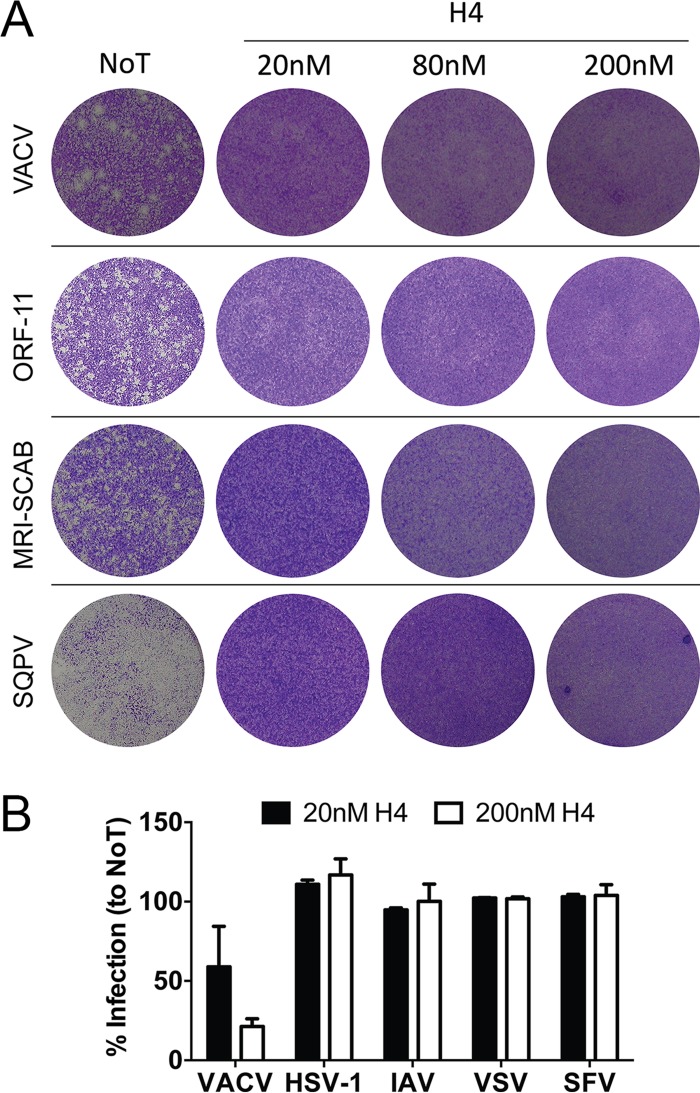
Poxviruses are acutely sensitive to H4 inhibitory activity. (A) Fetal lamb skin cell monolayers were infected with VACV or ORF-11 (100 PFU), MRI-SCAB (500 PFU), or squirrelpox virus (SQPV [1,000 PFU]). Cells were fixed and plaques visualized by crystal violet staining at 3 days (VACV), 4 days (ORF-11), or 7 days (MRI-SCAB and SQPV). Experiments were performed in triplicate; representative images are shown. (B) HeLa cells were infected with EGFP-expressing variants of VACV WR, herpes simplex virus 1 (HSV-1), influenza A virus (IAV), vesicular stomatitis virus (VSV), or Semliki Forest virus (SFV). For each, infection was allowed to proceed for 6 to 8 h, after which cells were analyzed for EGFP expression by flow cytometry. Experiments were performed in triplicate; the percentage of infection relative to untreated (NoT) controls is displayed as the mean ± SD.

Given the potent inhibitory effects of H4 on VACV transcription and replication, we asked if H4 could inhibit infection by other poxviruses. We tested three different parapoxviruses: ORF-11, MRI-SCAB (50), and squirrelpox virus (SQPV) (51). For this, fetal lamb skin cell monolayers were infected with these viruses in the presence of 20, 80, or 200 nM H4. Cell monolayers were assessed for plaque formation at 3 days p.i. with VACV or ORF-11 and 7 days with MRI-SCAB or SQPV. In the absence of H4, all viruses produced plaques, or in the case of SQPV destroyed the monolayer (Fig. 7A). Strikingly, treatment with H4 at 20, 80, or 200 nM completely attenuated plaque formation by all of the viruses (Fig. 7A). These results demonstrate that the bisbenzimide H4 is a broad-range inhibitor of poxvirus infection across different genera.

While we had shown previously that H4 did not affect plaque formation by human adenovirus (52), we wanted to test the impact of H4 on other viruses, including herpes simplex virus 1 (HSV-1), which replicates in the nucleus (53), and RNA viruses, which replicate either in the nucleus (influenza A virus [IAV]) or the cytoplasm (vesicular stomatitis virus [VSV] or Semliki Forest virus [SFV]). When cells were infected with these viruses in the presence of 20 or 200 nM H4, expression of GFP from reporter viruses was not significantly impacted regardless of the concentration used (Fig. 7B). We conclude that bisbenzimides are rather selective inhibitors against poxviruses and do not affect a broad range of unrelated viruses.

## DISCUSSION

Bisbenzimides are a class of fluorescent dyes commonly used in flow cytometry and fluorescence microscopy to identify cell nuclei. Bisbenzimides stain DNA in the nucleus and cytoplasmic organelles, such as mitochondria and chloroplasts (36). Here we demonstrate that a range of bisbenzimides are potent inhibitors of poxvirus infection. We used a variety of virological assays to determine the stage of the VACV life cycle impacted by H4, the most effective Hoechst derivative tested. We found that H4 did not inhibit infection by acting on extracellular viral particles or by targeting the early stages of the virus life cycle, including EGE and DNA uncoating. Analysis of viral early gene (EG), IG, and LG transcription, DNA replication site formation, and viral DNA synthesis indicated that H4 blocked IG and LG transcription as well as DNA replication in a dose-dependent manner. H4 strongly inhibited the production of infectious VACV particles, and plaque formation was impeded in its presence.

Our results indicate that the effectiveness of bisbenzimides against poxviruses correlates with the accessibility of the viral DNA to solutes. We found that the Hoechst compounds did not affect the infectivity of VACV particles when the particles were intact—that is, when they were outside cells. The compounds were effective after viral DNA was released from the capsid into the cytosol, where it is replicated. Consistent with this, the EC_50_ of H4 against EGE, which occurs when the viral core is largely intact, was 20-fold higher than the EC_50_ for IGE and LGE, which only occurs from exposed viral DNA. EG transcription occurs within cytoplasmic viral cores prior to genome uncoating, while intermediate and late gene transcription occurs in the cytoplasm after viral genome replication (54). High concentrations of H4 could impact EGE during the activation of cytosolic cores, which expand when EG transcription occurs (55–58). We speculate that core expansion may lead to increased accessibility of the VACV genomes within. That early, intermediate, and late VACV mRNAs are all translated in the cytoplasm on host ribosomes makes it unlikely that bisbenzimides impact translation of viral proteins.

We noted that the inhibitory efficacy of the bisbenzimides correlated with their lipophilicity: H4 > H8 > H5 (Fig. 1A). Lipophilicity largely dictates the binding of bisbenzimides to double-stranded DNA via hydrophobic interactions with adenosine-threonine (A = T)-rich regions (25, 59, 60). Since poxviruses with differential genomic A = T content—that is VACV (67% A = T), ORFV (36% A = T), and SQPV (33% A = T) (61)—display similar sensitivities to H4, it is unlikely that poxviruses are susceptible to bisbenzimides simply due to their high A = T content. It is more likely that the solute accessibility of the viral genome and the association of DNA binding proteins dictate susceptibility of the virus to the Hoechst compounds.

Remarkably, despite their effectiveness against poxviruses the Hoechst bisbenzimides tested had no effect on infection by other DNA viruses, such as adenovirus or herpesvirus. As opposed to poxviruses, herpesviruses and adenoviruses deliver their infectious incoming DNA genomes directly into the nucleus, where they are transcribed and replicated (48, 53, 62–64). It is possible that bisbenzimides do not efficiently bind to the nuclear DNA of adenovirus and herpesvirus due to the spatial proximity of host DNA, which acts as an efficient local avidity trap for the bisbenzimides. In this scenario, bisbenzimides used at low nanomolar, nontoxic concentrations would be more likely to bind to host DNA than viral DNA.

While selection of VACV variants resistant to either of the antipoxvirus agents cidofovir and ST-246 is readily possible (16, 65), we were unable to isolate a bisbenzimide-resistant VACV in up to 20 passages at different concentrations of H4 (data not shown). This is consistent with our finding that H4 targets at least two viral processes. Low concentrations of H4 inhibited IG and LG transcription, while higher concentrations of H4 also blocked viral DNA replication. IG and LG transcription inhibition at low H4 concentrations could occur since the intermediate and late promoters are AT rich (66), and H4 preferentially binds to AT-rich dsDNA. The presence of small highly condensed viral DNA replication sites observed in the presence of high concentrations of H4 and the observation that VACV topoisomerase DNA unwinding activity is unaffected *in vitro* (data not shown) (67) suggest a model in which bisbenzimides block DNA replication by coating cytoplasmic VACV genomes.

In sum, we show that bisbenzimide compounds are highly specific for inhibiting poxvirus infections at low apparent cytotoxicity. It is possible that the bisbenzimides tested here are also effective against divergent members of the nucleocytoplasmic large DNA viruses that replicate exclusively in the cytoplasm (68). Bisbenzimide compounds have been used in mice with potential antitumor effects (30) and were tested in a phase I-phase II advanced pancreatic carcinoma study in humans (69). Notably, in both cases bisbenzimides were well tolerated. While the *in vivo* efficacy of bisbenzimides against poxvirus infection has not been determined, the dual mechanism of inhibition—that is, I/L gene expression and viral DNA replication—appears to be a high barrier against the emergence of viral resistance. This makes it tempting to speculate that bisbenzimides may serve as attractive antipoxvirus drugs, either alone or in combination with CMX001 and ST-246 (70).

## MATERIALS AND METHODS

### Cell culture and reagents.

All cell lines used were cultivated as monolayers at 37.0°C and 5.0% CO_2_. Cells were cultured in Dulbecco's modified Eagle's medium (DMEM [Gibco, Life Technologies, Switzerland]). HeLa cells (ATCC) and L929 mouse subcutaneous areolar and adipose cells (ATCC) were cultivated in DMEM with the addition of 10% fetal bovine serum (FBS [Sigma]), 2 mM GlutaMAX (Life Technologies), and 1% penicillin-streptomycin (Pen-Strep [Sigma]). Cercopithecus aethiops kidney epithelial cells (BSC40; ATCC) were cultivated in DMEM with 10% FBS, 2 mM GlutaMAX, 1% nonessential amino acid mix (NEAA [Sigma]), and 1 mM sodium pyruvate (NaPyr [Sigma]). Cells of the HDFn human foreskin fibroblast cell line (Invitrogen) were cultivated in DMEM containing 5% FBS. Fetal lamb skin cells were cultivated in medium 199 (Sigma) with 2% glutamine, 0.16% sodium hydrogen carbonate, 10% tryptose phosphate broth, and 10% FBS.

### VACV and parapoxvirus strains and virus purification.

Vaccinia virus strain Western Reserve (VACV WR) was used throughout (71, 72). These strains were either wild type (WT) or transgenic containing early/late EGFP (E/L EGFP VACV WR), early EGFP (E EGFP VACV WR), intermediate EGFP (I EGFP VACV WR), or late EGFP (L EGFP VACV WR). All VACV mature virions (MVs) were purified from cytoplasmic lysates by being pelleted through a 36% sucrose cushion for 90 min at 18,000 × *g*. The virus pellet was resuspended in 10 mM Tris (pH 9.0) and subsequently banded on a 25 to 40% sucrose gradient at 14,000 × *g* for 45 min. Following centrifugation, the viral band was collected by aspiration and concentrated by pelleting at 14,000 × *g* for 45 min. MVs were resuspended in 1 mM Tris (pH 9.0), and the titer was determined for PFU per milliliter as previously described (73). The parapoxvirus strains used include a tissue culture-adapted strain, ORF-11, a nonadapted strain, MRI-SCAB, and squirrelpox virus (SQPV). IAV was obtained from Yohei Yamauchi, SFV and VSV were obtained from Giuseppe Balistreri, and HSV-1 was obtained from Cornel Fraefel.

### Inhibitors, dyes, antibodies, and plasmids.

Cycloheximide (CHX [Sigma]) was used at 50 μM, cytosine arabinoside (cytarabine, or AraC [Sigma]) was used at 10 μM. Bisbenzimides H4, H8, and H5 (Sigma) were dissolved in water and used as described in the respective experiments. Rabbit polyclonal anti-EGFP was used at a 1:1,000 dilution. Anti-I3 antibody (generously provided by Jakomine Krijnse Locker; Institute Pasteur) was used at 1:500. All secondary antibodies (goat anti-rabbit-AF488 and goat anti-rabbit-AF594 [Invitrogen]) were used at 1:1,000.

### Plaque 2.0 assay.

BSC40 cells were cultivated as monolayers in 96-well imaging plates (Greiner Bio-One, Germany) and inoculated with a serial dilution of either E/L EGFP VACV WR or E/L EGFP VACV IHD-J. One hour postinfection, the inoculum was removed and replaced with medium (nontreated control) or a respective dilution of an experimental compound in the medium. Twenty-four hours postinfection, plates were fixed with 4% paraformaldehyde (PFA) and stained with Hoechst nuclear stain. Plates were imaged using ImageXpress XL Micro epifluorescent high-throughput microscope (Molecular Devices, USA) with a 4× air objective (Nikon, Japan) in a tile mode allowing full well reconstruction. Image processing and analysis were performed using Plaque 2.0 software (41). The experiment was performed in three technical replicas (triplicate).

### Early, intermediate, and late VACV gene expression analysis.

HeLa or BSC40 cells in 24-well plates were infected with E EGFP VACV WR and L EGFP VACV WR at a multiplicity of infection (MOI) of 5 together with H4 at different concentrations. To quantify EGE or IGE and LGE, cells were infected for 6 or 8 h, respectively. Cells were detached with 0.05% trypsin-EDTA and fixed in 4% formaldehyde for 15 min. After centrifugation at 500 × *g* for 5 min, the cell pellets were resuspended in 400 μl fluorescence-activated cell sorter (FACS) buffer. A BD Bioscience FACSCalibur flow cytometer was used for analysis, and 10,000 cells per condition were measured.

### Virus titrations by plaque assay.

Viruses were diluted in the medium appropriate for the cell line, and 250 or 500 μl of this virus dilution was added to HeLa or fetal lamb skin cell monolayers in 6-well plates. The plates were rocked every 15 min, and after 1 h, medium was aspirated and cells fed with full medium containing the indicated compound concentrations. The cells were then incubated at 37°C for 3, 4, or 7 days (as indicated) before undergoing staining with 0.1% crystal violet in 3.7% PFA.

### Twenty-four-hour virus yield.

HeLa cells in 12-well plates were infected at an MOI of 1 in the presence of the compound. After 24 h, cells were collected and centrifuged, and the pellet was resuspended in 100 μl 1 mM Tris (pH 9.0). Cells were freeze-thawed three times to lyse the cells, and the virus solution was subjected to serial titration to determine the PFU per milliliter.

### Add-in and washout assays.

HeLa cells were grown in 12-well plates and infected with WT VACV WR at an MOI of 1. For washout experiments, infection was performed in the presence of 80 nM H4. At the indicated time point, the cells were washed five times with medium and infection was allowed to proceed in the absence of compound. For add-in experiments, medium containing 80 nM H4 was added to the cells at the indicated time points. For both experiments, at 24 hpi, cells were collected and titers determined as described above.

### Pretreatment of virus particles with H4.

Viruses expressing EGFP from early or late promoters were preincubated with 20 or 80 nM H4 for the indicated times at room temperature. The virus particles were washed three times in medium and used to infect HeLa cells. Similarly to early and late gene profiling, infection was stopped after 6 to 8 hpi, and infection was analyzed by flow cytometry.

### Prereplication site visualization.

HeLa cells were infected with WT VACV at an MOI of 10 in the presence of AraC and 20, 80, or 200 nM H4. At 5 hpi, cells were fixed, nuclei stained with DAPI, and prereplication sites visualized by immunofluorescence staining against VACV I3. Images were acquired by confocal microscopy, and maximum projections were generated from 10 z-stacks. The number of prereplication sites per cell in the presence of 20, 80, or 200 nM H4 was determined by spot detection of the maximum intensity projections (spots larger than 5 pixels) with the Fiji platform. Cells treated with CHX and AraC alone served as controls for genome uncoating and replication site formation, respectively.

### Replication site formation.

HeLa cells were infected with WT VACV at an MOI of 5 in the presence of 20, 80, or 200 nM H4. At 8 hpi, cells were fixed and VACV replication sites visualized by staining with DAPI. The percentage of cells containing replication sites was quantified by manual counting due to the shape and size variation of replication sites under the various conditions. AraC sample served as a control for inhibition of DNA replication site formation.

### EdU accumulation.

HeLa cells were infected with WT VACV at an MOI of 10. After 1 h of virus binding in DMEM, the medium was changed to 10% DMEM containing 1 μM EdU in the presence or absence of H4 (20, 80, or 200 nM). At 8 hpi, cells were fixed with 4% PFA and stained using the Click-iT EdU imaging kit (Thermo Scientific) and Hoechst to visualize cell nuclei. Cells were imaged using confocal microscopy and analyzed using CellProfiler/KNIME software. Briefly, the intensity of EdU staining per sample was determined after background subtraction and exclusion of nuclei by image segmentation.

### Viral DNA quantification by qPCR.

HeLa cell monolayers were infected with WR (MOI of 10) in the absence or presence of H4 for 8 h. Total DNA was extracted using the Qiagen DNeasy blood and tissue kit according to the manufacturer's instruction. Total DNA concentrations were assessed using a Nanodrop spectrophotometer, and a portion of total DNA was used in the qPCR assay using Mesa Blue qPCR MasterMix Plus for the SYBR assay system (Eurogentec) with the C11R primer (5′-AAACACACACTGAGAAACAGCATAAA-3′ and 5′-ACTATCGGCGAATGATCTGATTA-3′). The concentration of viral DNA was determined by plotting against a standard curve of VACV DNA from purified virions.

### RT-PCR.

HeLa cell monolayers were infected with WR (MOI of 10) in the absence or presence of H4 for 2, 4, or 8 h. Total RNA was harvested from infected cells using the Qiagen RNeasy kit according to the manufacturer's instructions. Subsequently, 1 μl of total RNA was reverse transcribed into single-stranded cDNA with SuperScript II reverse transcriptase (Thermo Fisher Scientific) and oligo(dT) primers. Amplification of J2 (early) from 2-h samples, G8 (intermediate) from 4-h samples, F17 (late) from 8-h samples, and glyceraldehyde-3-phosphate dehydrogenase (GAPDH) cDNA from all time points was performed by qPCR (Mesa Blue qPCR MasterMix Plus for SYBR assay; Eurogentec) using primers specific for VACV J2R (5′-TACGGAACGGGACTATGGAC-3′ and 5′-GTTTGCCATACGCTCACAGA-3′), G8R (5′-AATGTAGACTCGACGGATGAGTTA-3′ and 5′-TCGTCATTATCCATTACGATTCTAGTT-3′), F17R (5′-ATTCTCATTTTGCATCTGCTC-3′ and 5′-AGCTACATTATCGCGATTAGC-3′), and GAPDH (5′ AAGGTCGGAGTCAACGGATTTGGT-3′ and 5′-ACAAAGTGGTCGTTGAGGGCAATG-3′). Viral mRNA threshold cycle (*C_T_*) values are displayed as abundance normalized against GAPDH.

### Influenza A virus, Semliki Forest virus, vesicular stomatitis virus, and herpes simplex virus 1 infections.

EGFP-expressing variants of the indicated viruses were used for these experiments. For each, HeLa cells were infected at an MOI of 5 in the presence of 20 or 200 nM H4 and cells were prepared for flow cytometry analysis between 6 and 8 hpi.

## References

[1] Lederberg J 2000 Infectious history. Science 288:287–293. doi:10.1126/science.288.5464.287. 10777411

[2] Greber UF, Bartenschlager R 2017 Editorial: an expanded view of viruses. FEMS Microbiol Rev 41:1–4. doi:10.1093/femsre/fuw044. 28087690 PMC7108522

[3] Koonin EV, Dolja VV, Krupovic M 2015 Origins and evolution of viruses of eukaryotes: the ultimate modularity. Virology 479:2–25. doi:10.1016/j.virol.2015.02.039. 25771806 PMC5898234

[4] Prasad V, Suomalainen M, Hemmi S, Greber UF 2017 The cell cycle-dependent kinase Cdk9 is a postexposure drug target against human adenoviruses. ACS Infect Dis 3:398–405. doi:10.1021/acsinfecdis.7b00009. 28434229

[5] Santos-Fernandes E, Beltrame CO, Byrd CM, Cardwell KB, Schnellrath LC, Medaglia ML, Hruby DE, Jordan R, Damaso CR 2013 Increased susceptibility of Cantagalo virus to the antiviral effect of ST-246. Antiviral Res 97:301–311. doi:10.1016/j.antiviral.2012.11.010. 23257396

[6] Moussatche N, Damaso CR, McFadden G 2008 When good vaccines go wild: feral orthopoxvirus in developing countries and beyond. J Infect Dev Ctries 2:156–173. doi:10.3855/jidc.258. 19738346

[7] Kurth A, Wibbelt G, Gerber HP, Petschaelis A, Pauli G, Nitsche A 2008 Rat-to-elephant-to-human transmission of cowpox virus. Emerg Infect Dis 14:670–671. doi:10.3201/eid1404.070817. 18394293 PMC2570944

[8] Vogel S, Sardy M, Glos K, Korting HC, Ruzicka T, Wollenberg A 2012 The Munich outbreak of cutaneous cowpox infection: transmission by infected pet rats. Acta Derm Venereol 92:126–131. doi:10.2340/00015555-1227. 22041995

[9] Duraffour S, Mertens B, Meyer H, van den Oord JJ, Mitera T, Matthys P, Snoeck R, Andrei G 2013 Emergence of cowpox: study of the virulence of clinical strains and evaluation of antivirals. PLoS One 8:e55808. doi:10.1371/journal.pone.0055808. 23457480 PMC3574090

[10] Di Giulio DB, Eckburg PB 2004 Human monkeypox: an emerging zoonosis. Lancet Infect Dis 4:15–25. doi:10.1016/S1473-3099(03)00856-9. 14720564 PMC9628772

[11] Hutson CL, Lee KN, Abel J, Carroll DS, Montgomery JM, Olson VA, Li Y, Davidson W, Hughes C, Dillon M, Spurlock P, Kazmierczak JJ, Austin C, Miser L, Sorhage FE, Howell J, Davis JP, Reynolds MG, Braden Z, Karem KL, Damon IK, Regnery RL 2007 Monkeypox zoonotic associations: insights from laboratory evaluation of animals associated with the multi-state US outbreak. Am J Trop Med Hyg 76:757–768. 17426184

[12] Shchelkunov SN 2013 An increasing danger of zoonotic orthopoxvirus infections. PLoS Pathog 9:e1003756. doi:10.1371/journal.ppat.1003756. 24339772 PMC3855571

[13] Kemper AR, Davis MM, Freed GL 2002 Expected adverse events in a mass smallpox vaccination campaign. Eff Clin Pract 5:84–90. 11990216

[14] Belongia EA, Naleway AL 2003 Smallpox vaccine: the good, the bad, and the ugly. Clin Med Res 1:87–92. doi:10.3121/cmr.1.2.87. 15931293 PMC1069029

[15] De Clercq E 2002 Cidofovir in the treatment of poxvirus infections. Antiviral Res 55:1–13. doi:10.1016/S0166-3542(02)00008-6. 12076747 PMC9533828

[16] Yang G, Pevear DC, Davies MH, Collett MS, Bailey T, Rippen S, Barone L, Burns C, Rhodes G, Tohan S, Huggins JW, Baker RO, Buller RL, Touchette E, Waller K, Schriewer J, Neyts J, DeClercq E, Jones K, Hruby D, Jordan R 2005 An orally bioavailable antipoxvirus compound (ST-246) inhibits extracellular virus formation and protects mice from lethal orthopoxvirus challenge. J Virol 79:13139–13149. doi:10.1128/JVI.79.20.13139-13149.2005. 16189015 PMC1235851

[17] Blasco R, Moss B 1991 Extracellular vaccinia virus formation and cell-to-cell virus transmission are prevented by deletion of the gene encoding the 37,000-dalton outer envelope protein. J Virol 65:5910–5920. 1920620 10.1128/jvi.65.11.5910-5920.1991 PMC250254

[18] Chinsangaram J, Honeychurch KM, Tyavanagimatt SR, Leeds JM, Bolken TC, Jones KF, Jordan R, Marbury T, Ruckle J, Mee-Lee D, Ross E, Lichtenstein I, Pickens M, Corrado M, Clarke JM, Frimm AM, Hruby DE 2012 Safety and pharmacokinetics of the anti-orthopoxvirus compound ST-246 following a single daily oral dose for 14 days in human volunteers. Antimicrob Agents Chemother 56:4900–4905. doi:10.1128/AAC.00904-12. 22777041 PMC3421894

[19] Becker MN, Obraztsova M, Kern ER, Quenelle DC, Keith KA, Prichard MN, Luo M, Moyer RW 2008 Isolation and characterization of cidofovir resistant vaccinia viruses. Virol J 5:58. doi:10.1186/1743-422X-5-58. 18479513 PMC2397383

[20] James SH, Price NB, Hartline CB, Lanier ER, Prichard MN 2013 Selection and recombinant phenotyping of a novel CMX001 and cidofovir resistance mutation in human cytomegalovirus. Antimicrob Agents Chemother 57:3321–3325. doi:10.1128/AAC.00062-13. 23650158 PMC3697342

[21] De Gascun CF, Carr MJ 2013 Human polyomavirus reactivation: disease pathogenesis and treatment approaches. Clin Dev Immunol 2013:373579. doi:10.1155/2013/373579. 23737811 PMC3659475

[22] Magee WC, Hostetler KY, Evans DH 2005 Mechanism of inhibition of vaccinia virus DNA polymerase by cidofovir diphosphate. Antimicrob Agents Chemother 49:3153–3162. doi:10.1128/AAC.49.8.3153-3162.2005. 16048917 PMC1196213

[23] Bradbury J 2002 Orally available cidofovir derivative active against smallpox. Lancet 359:1041. doi:10.1016/S0140-6736(02)08115-1. 11937193

[24] Morris K 1998 Short course of AZT halves HIV-1 perinatal transmission. Lancet 351:651. doi:10.1016/S0140-6736(05)78436-1. 9500334

[25] Fornander LH, Wu L, Billeter M, Lincoln P, Norden B 2013 Minor-groove binding drugs: where is the second Hoechst 33258 molecule? J Phys Chem B 117:5820–5830. doi:10.1021/jp400418w. 23607615

[26] Han F, Taulier N, Chalikian TV 2005 Association of the minor groove binding drug Hoechst 33258 with d(CGCGAATTCGCG)2: volumetric, calorimetric, and spectroscopic characterizations. Biochemistry 44:9785–9794. doi:10.1021/bi047374f. 16008363

[27] Smith PJ, Lacy M, Debenham PG, Watson JV 1988 A mammalian cell mutant with enhanced capacity to dissociate a bis-benzimidazole dye-DNA complex. Carcinogenesis 9:485–490. doi:10.1093/carcin/9.3.485. 3345586

[28] Comings D, Avelino E 1975 Mechanisms of chromosome banding. Chromosoma 51:365–379. doi:10.1007/BF00326323. 51710

[29] Latt SA, Stetten G 1976 Spectral studies on 33258 Hoechst and related bisbenzimidazole dyes useful for fluorescent detection of deoxyribonucleic acid synthesis. J Histochem Cytochem 24:24–33. doi:10.1177/24.1.943439. 943439

[30] Olive PL, Chaplin DJ, Durand RE 1985 Pharmacokinetics, binding and distribution of Hoechst 33342 in spheroids and murine tumours. Br J Cancer 52:739–746. doi:10.1038/bjc.1985.252. 4063148 PMC1977212

[31] Wang X-J, Chu N-Y, Wang Q-H, Liu C, Jiang C-G, Wang X-Y, Ikejima T, Cheng M-S 2012 Newly synthesized bis-benzimidazole derivatives exerting anti-tumor activity through induction of apoptosis and autophagy. Bioorg Med Chem Lett 22:6297–6300. doi:10.1016/j.bmcl.2012.06.102. 22959518

[32] Baraldi PG, Bovero A, Fruttarolo F, Preti D, Tabrizi MA, Pavani MG, Romagnoli R 2004 DNA minor groove binders as potential antitumor and antimicrobial agents. Med Res Rev 24:475–528. doi:10.1002/med.20000. 15170593

[33] Chen AY, Yu C, Bodley A, Peng LF, Liu LF 1993 A new mammalian DNA topoisomerase I poison Hoechst 33342: cytotoxicity and drug resistance in human cell cultures. Cancer Res 53:1332–1337. 8383008

[34] Soderlind KJ, Gorodetsky B, Singh AK, Bachur NR, Miller GG, Lown JW 1999 Bis-benzimidazole anticancer agents: targeting human tumour helicases. Anticancer Drug Des 14:19–36. 10363025

[35] Woynarowski JM, McHugh M, Sigmund RD, Beerman TA 1989 Modulation of topoisomerase II catalytic activity by DNA minor groove binding agents distamycin, Hoechst 33258, and 4′,6-diamidine-2-phenylindole. Mol Pharmacol 35:177–182. 2465485

[36] Latt SA, Stetten G, Juergens LA, Willard HF, Scher CD 1975 Recent developments in the detection of deoxyribonucleic acid synthesis by 33258 Hoechst fluorescence. J Histochem Cytochem 23:493–505. doi:10.1177/23.7.1095650. 1095650

[37] Downs TR, Wilfinger WW 1983 Fluorometric quantification of DNA in cells and tissue. Anal Biochem 131:538–547. doi:10.1016/0003-2697(83)90212-9. 6193739

[38] Goracci L, Germani R, Savelli G, Bassani DM 2005 Hoechst 33258 as a pH-sensitive probe to study the interaction of amine oxide surfactants with DNA. Chembiochem 6:197–203. doi:10.1002/cbic.200400196. 15549726

[39] Schabel FM Jr 1968 The antiviral activity of 9-β-d-arabinofuranosyladenine (ARA-A). Chemotherapy 13:321–338. doi:10.1159/000220567. 4305653

[40] Furth JJ, Cohen SS 1968 Inhibition of mammalian DNA polymerase by the 5′-triphosphate of 1-beta-d-arabinofuranosylcytosine and the 5′-triphosphate of 9-beta-d-arabinofuranoxyladenine. Cancer Res 28:2061–2067. 5754840

[41] Yakimovich A, Andriasyan V, Witte R, Wang IH, Prasad V, Suomalainen M, Greber UF 2015 Plaque2.0—a high-throughput analysis framework to score virus-cell transmission and clonal cell expansion. PLoS One 10:e0138760. doi:10.1371/journal.pone.0138760. 26413745 PMC4587671

[42] Kilcher S, Mercer J 2015 DNA virus uncoating. Virology 479:578–590. doi:10.1016/j.virol.2015.01.024. 25728300

[43] Yamauchi Y, Greber UF 2016 Principles of virus uncoating: cues and the snooker ball. Traffic 17:569–592. doi:10.1111/tra.12387. 26875443 PMC7169695

[44] Mercer J, Snijder B, Sacher R, Burkard C, Bleck CK, Stahlberg H, Pelkmans L, Helenius A 2012 RNAi screening reveals proteasome- and Cullin3-dependent stages in vaccinia virus infection. Cell Rep 2:1036–1047. doi:10.1016/j.celrep.2012.09.003. 23084750

[45] Welsch S, Doglio L, Schleich S, Krijnse Locker J 2003 The vaccinia virus I3L gene product is localized to a complex endoplasmic reticulum-associated structure that contains the viral parental DNA. J Virol 77:6014–6028. doi:10.1128/JVI.77.10.6014-6028.2003. 12719593 PMC154049

[46] Artenstein AW, Johnson C, Marbury TC, Morrison D, Blum PS, Kemp T, Nichols R, Balser JP, Currie M, Monath TP 2005 A novel, cell culture-derived smallpox vaccine in vaccinia-naive adults. Vaccine 23:3301–3309. doi:10.1016/j.vaccine.2005.01.079. 15837236

[47] Kilcher S, Schmidt FI, Schneider C, Kopf M, Helenius A, Mercer J 2014 siRNA screen of early poxvirus genes identifies the AAA+ ATPase D5 as the virus genome-uncoating factor. Cell Host Microbe 15:103–112. doi:10.1016/j.chom.2013.12.008. 24439902

[48] Wang I-H, Suomalainen M, Andriasyan V, Kilcher S, Mercer J, Neef A, Luedtke NW, Greber UF 2013 Tracking viral genomes in host cells at single-molecule resolution. Cell Host Microbe 14:468–480. doi:10.1016/j.chom.2013.09.004. 24139403

[49] Senkevich TG, Koonin EV, Moss B 2009 Predicted poxvirus FEN1-like nuclease required for homologous recombination, double-strand break repair and full-size genome formation. Proc Natl Acad Sci U S A 106:17921–17926. doi:10.1073/pnas.0909529106. 19805122 PMC2755464

[50] McInnes CJ, Wood AR, Nettleton PF, Gilray JA 2001 Genomic comparison of an avirulent strain of Orf virus with that of a virulent wild type isolate reveals that the Orf virus G2L gene is non-essential for replication. Virus Genes 22:141–150. doi:10.1023/A:1008117127729. 11324750

[51] McInnes CJ, Wood AR, Thomas K, Sainsbury AW, Gurnell J, Dein FJ, Nettleton PF 2006 Genomic characterization of a novel poxvirus contributing to the decline of the red squirrel (Sciurus vulgaris) in the UK. J Gen Virol 87:2115–2125. doi:10.1099/vir.0.81966-0. 16847106

[52] Yakimovich A, Gumpert H, Burckhardt CJ, Lütschg VA, Jurgeit A, Sbalzarini IF, Greber UF 2012 Cell-free transmission of human adenovirus by passive mass transfer in cell culture simulated in a computer model. J Virol 86:10123–10137. doi:10.1128/JVI.01102-12. 22787215 PMC3446567

[53] Flatt JW, Greber UF 12 5 2017 Viral mechanisms for docking and delivering at nuclear pore complexes. Semin Cell Dev Biol doi:10.1016/j.semcdb.2017.05.008. 28506891

[54] Moss B 2007 Poxviridae: the viruses and their replication, p 2905–2946. *In* Knipe DM, Howley PM, Griffin DE, Lamb RA, Martin MA, Roizman B, Straus SE (ed), Fields virology, 5th ed Lippincott Williams & Wilkins, Philadelphia, PA.

[55] Cyrklaff M, Risco C, Fernandez JJ, Jimenez MV, Esteban M, Baumeister W, Carrascosa JL 2005 Cryo-electron tomography of vaccinia virus. Proc Natl Acad Sci U S A 102:2772–2777. doi:10.1073/pnas.0409825102. 15699328 PMC549483

[56] Dales S 1963 The uptake and development of vaccinia virus in strain L cells followed with labeled viral deoxyribonucleic acid. J Cell Biol 18:51–72. doi:10.1083/jcb.18.1.51. 14024720 PMC2106286

[57] Hollinshead M, Vanderplasschen A, Smith GL, Vaux DJ 1999 Vaccinia virus intracellular mature virions contain only one lipid membrane. J Virol 73:1503–1517. 9882356 10.1128/jvi.73.2.1503-1517.1999 PMC103975

[58] Schmidt FI, Bleck CK, Reh L, Novy K, Wollscheid B, Helenius A, Stahlberg H, Mercer J 2013 Vaccinia virus entry is followed by core activation and proteasome-mediated release of the immunomodulatory effector VH1 from lateral bodies. Cell Rep 4:464–476. doi:10.1016/j.celrep.2013.06.028. 23891003

[59] Chang DK, Cheng SF 1996 On the importance of van der Waals interaction in the groove binding of DNA with ligands: restrained molecular dynamics study. Int J Biol Macromol 19:279–285. doi:10.1016/S0141-8130(96)01138-5. 9024904

[60] Sauers RR 1995 An analysis of van der Waals attractive forces in DNA-minor groove binding. Bioorg Med Chem Lett 5:2573–2576. doi:10.1016/0960-894X(95)00438-Y.

[61] Hatcher EL, Wang C, Lefkowitz EJ 2015 Genome variability and gene content in chordopoxviruses: dependence on microsatellites. Viruses 7:2126–2146. doi:10.3390/v7042126. 25912716 PMC4411693

[62] Flatt JW, Greber UF 2015 Misdelivery at the nuclear pore complex—stopping a virus dead in its tracks. Cells 4:277–296. doi:10.3390/cells4030277. 26226003 PMC4588037

[63] Kobiler O, Drayman N, Butin-Israeli V, Oppenheim A 2012 Virus strategies for passing the nuclear envelope barrier. Nucleus 3:526–539. doi:10.4161/nucl.21979. 22929056 PMC3515536

[64] Puntener D, Greber UF 2009 DNA-tumor virus entry—from plasma membrane to the nucleus. Semin Cell Dev Biol 20:631–642. 19588547 10.1016/j.semcdb.2009.03.014

[65] Smee DF, Sidwell RW, Kefauver D, Bray M, Huggins JW 2002 Characterization of wild-type and cidofovir-resistant strains of camelpox, cowpox, monkeypox, and vaccinia viruses. Antimicrob Agents Chemother 46:1329–1335. doi:10.1128/AAC.46.5.1329-1335.2002. 11959564 PMC127179

[66] Moss B 2013 Poxviridae, p 2129–2159. *In* Fields BN, Knipe DM, Howley PM, Cohen IJ, Griffin DE, Lamb RA, Martin MA, Roizman B, Straus SE (ed), Fields virology, 6th ed, vol 2 Lippincott Williams & Wilkins, Philadelphia, PA.

[67] Sekiguchi J, Shuman S 1997 Mutational analysis of vaccinia virus topoisomerase identifies residues involved in DNA binding. Nucleic Acids Res 25:3649–3656. doi:10.1093/nar/25.18.3649. 9278486 PMC146948

[68] Mutsafi Y, Fridmann-Sirkis Y, Milrot E, Hevroni L, Minsky A 2014 Infection cycles of large DNA viruses: emerging themes and underlying questions. Virology 466-467:3–14. doi:10.1016/j.virol.2014.05.037. 24996494

[69] Patel SR, Kvols LK, Rubin J, O'Connell MJ, Edmonson JH, Ames MM, Kovach JS 1991 Phase I-II study of pibenzimol hydrochloride (NSC 322921) in advanced pancreatic carcinoma. Invest New Drugs 9:53–57. doi:10.1007/BF00194545. 1709152

[70] Quenelle DC, Prichard MN, Keith KA, Hruby DE, Jordan R, Painter GR, Robertson A, Kern ER 2007 Synergistic efficacy of the combination of ST-246 with CMX001 against orthopoxviruses. Antimicrob Agents Chemother 51:4118–4124. doi:10.1128/AAC.00762-07. 17724153 PMC2151443

[71] Mercer J, Helenius A 2008 Vaccinia virus uses macropinocytosis and apoptotic mimicry to enter host cells. Science 320:531–535. doi:10.1126/science.1155164. 18436786

[72] Mercer J, Knebel S, Schmidt FI, Crouse J, Burkard C, Helenius A 2010 Vaccinia virus strains use distinct forms of macropinocytosis for host-cell entry. Proc Natl Acad Sci U S A 107:9346–9351. doi:10.1073/pnas.1004618107. 20439710 PMC2889119

[73] Mercer J, Traktman P 2003 Investigation of structural and functional motifs within the vaccinia virus A14 phosphoprotein, an essential component of the virion membrane. J Virol 77:8857–8871. doi:10.1128/JVI.77.16.8857-8871.2003. 12885904 PMC167248

